# Polystyrene microplastics facilitate *Clostridioides difficile* biofilm formation and attenuate antibiotic susceptibility

**DOI:** 10.1128/aem.01168-26

**Published:** 2026-07-31

**Authors:** Xilong Deng, Chen Yang, Lu Chen, Ruijia Chen, Jingpeng Yang

**Affiliations:** 1 State Key Laboratory of Microbial Technology, School of Food Science and Pharmaceutical Engineering, Nanjing Normal University, Qixia 12534, Nanjing, Jiangsu, China; Indiana University Bloomington, Bloomington, Indiana, USA

**Keywords:** *Clostridioides difficile *infection, polystyrene microplastics, pathogenicity, biofilm, antibiotic resistance

## Abstract

*Clostridioides difficile* infection (CDI) constitutes a critical global public health challenge, with its high recurrence rates intrinsically linked to spore germination, biofilm formation, and antibiotic resistance. Although microplastics are recognized as emerging foodborne contaminants, their potential to exacerbate the risk of CDI recurrence remains largely unexplored. This study systematically elucidates the impact of polystyrene microplastics (PS-MPs) on *C. difficile* (CD) pathogenicity, biofilm dynamics, and antibiotic resistance. Exposure to PS-MPs (0–400 μg/mL) induced intracellular oxidative stress, thereby facilitating bacterial proliferation and biofilm formation. Concurrently, PS-MPs upregulated the expression of quorum-sensing genes (*agrD*, *luxS*) and enhanced AI-2 secretion, which subsequently augmented bacterial motility. Furthermore, PS-MPs exposure significantly elevated the expression of virulence and sporulation genes (e.g., *Spo0A*), intensifying cytotoxicity toward intestinal epithelial cells (HT-29 and Caco-2). Critically, antimicrobial susceptibility testing demonstrated that both short-term (48 h) and long-term (20 d) PS-MPs exposure significantly increased the half-inhibitory concentration (IC₅₀) of CD against seven antibiotics. Notably, long-term exposure to 100 μg/mL PS-MPs resulted in a 2.42-fold increase in the IC₅₀ for vancomycin, concomitant with the upregulation of resistance genes (*tetW*, *gyrA*, and *gyrB*). Collectively, these findings indicate that PS-MPs exposure potentiates CD pathogenicity and antibiotic resistance by activating the quorum-sensing system and facilitating biofilm formation. This study provides novel evidence linking environmental pollutants to CDI epidemiology, suggesting that microplastic pollution may compound the clinical recurrence risk and therapeutic challenges associated with CDI.

**IMPORTANCE:**

It is well established that both microplastics and *C. difficile* (CD) can enter the human body through the food chain, where they pose significant health risks. However, the mechanistic interactions between these two factors remain poorly understood. In this study, we provide novel insights into this interaction by demonstrating that polystyrene microplastics induce intracellular oxidative stress in CD, thereby activating quorum-sensing pathways and promoting biofilm formation. These events collectively enhance bacterial proliferation, motility, and virulence expression. More importantly, microplastic exposure substantially increases the tolerance of CD to multiple clinically relevant antibiotics, including vancomycin, an effect that is closely associated with the upregulation of key antibiotic resistance genes. Collectively, these findings reveal that environmental microplastic pollution not only serves as a physical vector for pathogen dissemination but also exacerbates the therapeutic challenges and recurrence risk associated with CD infections through direct modulation of bacterial pathogenicity and antimicrobial resistance.

## INTRODUCTION

In recent years, *Clostridioides difficile* infection (CDI) has emerged as the predominant cause of healthcare-associated diarrhea globally, with its escalating incidence and mortality posing a critical threat to public health (1). The pathogenesis of CDI is a multifaceted process encompassing gut colonization, biofilm formation, toxin production, and the dynamic transition between spore and vegetative states (2). While antibiotics remain the cornerstone of clinical management, their widespread overuse disrupts gut microbiota composition and compromises intestinal homeostasis, thereby creating a permissive niche for *C. difficile* (CD) colonization and proliferation (3, 4). Consequently, the proliferation of drug-resistant strains and persistently high recurrence rates has substantially undermined the efficacy of conventional therapeutic regimens (5). Notably, enhanced antibiotic resistance in CD has augmented its ecological fitness and transmission potential across both host and environmental reservoirs—a phenomenon driven by a complex interplay of determinants (6). Given the growing recognition of environmental triggers, elucidating the specific exogenous factors that exacerbate CD pathogenicity and accelerate resistance evolution is imperative for optimizing CDI prevention and control strategies.

As emerging environmental contaminants, microplastics have achieved ubiquitous distribution across global ecosystems. Substantial evidence confirms their prevalence in diverse food matrices and their potential for bioaccumulation in human organs via food chain transmission, thereby posing significant health risks, notably by serving as vectors for pathogens (7, 8). Previous investigations have demonstrated that microplastic exposure significantly enhances biofilm formation and antibiotic resistance in pathogens such as *Escherichia coli*, *Pseudomonas aeruginosa*, and *Vibrio cholerae*, consequently exacerbating infection risks (9–12). Furthermore, *in vivo* studies have elucidated the role of micro/nanoplastics in amplifying microbial pathogenicity. For instance, Maione et al. observed that exposure to polystyrene microplastics (PS-MPs) significantly increased the infectivity of *Candida albicans* in *Galleria mellonella* larvae and exacerbated inflammatory responses (13). Similarly, Zhang et al. confirmed that polystyrene nanoplastics promote *Helicobacter pylori* colonization and aggravate gastrointestinal tissue damage in mice (14). Although direct clinical evidence linking microplastic exposure to elevated human infection rates remains limited, existing preclinical data suggest that microplastics are reshaping the ecological traits and epidemiological patterns of pathogenic microorganisms, constituting a non-negligible threat. Notably, CD spores have recently been detected in various food products, including meat and vegetables, indicating a potential risk of foodborne transmission (15). In this context, microplastics—ubiquitous within the food chain—may play a pivotal role in the environmental dissemination and host infection of CD.

To the best of our knowledge, the impact of microplastic exposure on the biological characteristics of CD remains largely unexplored. Given that the high recurrence rate of CDI is primarily driven by spore-forming capacity and biofilm-mediated resistance, and recognizing the established role of microplastics as vectors for bacterial adhesion, we hypothesized that microplastic exposure potentiates the resistance and recurrence potential of CD. Accordingly, this study systematically evaluated the effects of PS-MPs across a gradient of concentrations on the physiological phenotypes and antibiotic resistance of CD. Initially, by characterizing the interactions between PS-MPs and CD, we assessed intracellular oxidative stress levels and cell membrane permeability to elucidate the physiological stress responses elicited by PS-MPs. Subsequently, we investigated the regulatory mechanisms underlying PS-MPs-induced biofilm formation and pathogenicity from a multidimensional perspective, encompassing quorum-sensing system regulation, bacterial motility, extracellular polymeric substance (EPS) secretion, sporulation capacity, and virulence gene expression. Finally, we characterized the evolutionary trajectory of antibiotic resistance in CD under PS-MPs stress. This study aims to elucidate the potential link between PS-MPs exposure and the pathogenesis of CDI, providing a scientific basis for developing targeted prevention and control strategies.

## MATERIALS AND METHODS

### Strains, cells, and reagents

*Clostridioides difficile* ATCC 43255 (VPI 10463, CD) and *Vibrio campbellii* ATCC BAA-1117 (VC) were obtained from the American Type Culture Collection (ATCC). CD was cultured anaerobically in Brain Heart Infusion (BHI) broth within an anaerobic workstation (ELACTROTEC; atmosphere: 10% H₂, 10% CO₂, 80% N₂) at 37°C. VC was cultivated in 2216E medium (Hopebio, Qingdao, China), adjusted to the target experimental concentration, and subsequently incubated in freshly prepared autoinducer (AB) broth (17.5 g/L NaCl, 12.3 g/L MgSO₄, 2.0 g/L acid-hydrolyzed casein, pH 7.5) at 30°C. Human colorectal adenocarcinoma cell lines, HT-29 and Caco-2, were preserved in our laboratory. HT-29 cells were maintained in RPMI-1640 complete medium, comprising RPMI-1640 basal medium (Kaiji Bio, Jiangsu, China) supplemented with 10% (vol/vol) fetal bovine serum (FBS; Gibco, Jiangsu, China) and 1% penicillin-streptomycin (100 IU/mL penicillin and 100 μg/mL streptomycin). Caco-2 cells were cultured in high-glucose Dulbecco’s Modified Eagle Medium (DMEM) supplemented with 20% (vol/vol) FBS (Meisen Cell, Zhejiang, China). Polystyrene microplastics (PS-MPs) with a particle size of 5 μm were purchased from Jiangsu Zhichuan Technology Co., Ltd. Polystyrene microplastic (PS-MPs) powder was suspended in ultrapure water to formulate a homogeneous stock suspension (25 mg/mL). Working solutions at target concentrations were subsequently prepared via precise serial dilutions of this stock, ensuring the accuracy of the final exposure doses. All suspensions were freshly prepared and vortexed immediately before use to maintain homogeneity during the experiments.

### Morphological and spectroscopic characterization of *C. difficile* exposed to PS-MPs

Initially, the morphology and microstructure of the PS-MPs were characterized using scanning electron microscopy (SEM; Apreo 2S, Thermo Scientific, USA), while their particle size distribution was determined using a nanoparticle size analyzer (Malvern Instruments, Malvern, UK). To elucidate the interactions between PS-MPs and CD, bacterial cultures were co-incubated with PS-MPs under anaerobic conditions for 48 h. The resulting precipitates were harvested via centrifugation (12,000 rpm, 20 min), with a CD monoculture processed concurrently as a control. Following lyophilization, the infrared absorption spectra (4,000–400 cm⁻¹) of the samples were acquired using a Fourier Transform Infrared Spectrometer (FT-IR; Nicolet 6700, Thermo Scientific, USA) (16). In addition, CD cells co-cultured with 100 μg/mL PS-MPs will be examined using confocal laser scanning microscopy (CLSM, AX Confocal Microscope System, Nikon, Japan) and scanning electron microscopy (SEM, Apreo 2S, Thermo Scientific, USA) to observe adsorption phenomena and physical interactions.

### Impact of PS-MPs on growth vitality, intracellular ROS accumulation, and membrane permeability of planktonic growth *C. difficile*

*C. difficile* (CD) was inoculated into BHI broth and cultured to the mid-exponential phase. Cells were harvested by centrifugation (4°C, 6,000 rpm, 10 min) and resuspended in fresh BHI broth to a standardized optical density at 600 nm (OD₆₀₀) of 0.2. Subsequently, 50 μL of the bacterial suspension was inoculated into fresh BHI medium supplemented with varying concentrations of PS-MPs (0, 10, 100, and 400 μg/mL). The cultures were incubated anaerobically at 37°C for 48 h, after which bacterial viability was determined using the standard plate count method.

Intracellular reactive oxygen species (ROS) levels and cell membrane permeability in planktonic growth CD cells were assessed following previously established protocols with minor modifications (17). Briefly, CD cells were prepared as described above, washed three times with phosphate-buffered saline (PBS), and adjusted to an OD₆₀₀ of 0.2. The bacterial suspension was then exposed to PS-MPs (0, 10, 100, and 400 μg/mL) and incubated at 37°C for 4 h. Intracellular ROS levels were quantified using a commercial ROS assay kit (Beyotime, S0034S, Shanghai, China). Cell membrane permeability was evaluated using propidium iodide (PI) staining. Following exposure, PI was added to the bacterial suspension at a final concentration of 10 μg/mL, followed by incubation in the dark at 37°C for 20 min. Fluorescence intensity was measured using a microplate reader with excitation (H1M, BioTek, USA) and emission wavelengths set at 535 nm and 615 nm, respectively.

### Impact of PS-MPs on biofilm formation by *C. difficile*

Biofilm formation was quantified using a crystal violet (CV) staining assay, adapted from a previously described method (18). Briefly, CD suspensions were inoculated into 24-well plates containing BHI medium supplemented with varying concentrations of PS-MPs (0, 10, 100, and 400 μg/mL). Each treatment was performed in triplicate. The plates were incubated anaerobically at 37°C for 72 h to facilitate biofilm development. Post-incubation, the culture supernatant was discarded, and the wells were gently rinsed three times with PBS to remove planktonic bacteria. The adherent biofilms were air-dried at room temperature, fixed with 200 μL of methanol for 20 min, and rinsed again with PBS. Subsequently, the biofilms were stained with 200 μL of 0.1% (wt/vol) crystal violet solution for 30 min. Excess stain was removed by washing with PBS, and the plates were air-dried. Finally, the bound dye was solubilized in 200 μL of 33% (vol/vol) glacial acetic acid with gentle shaking for 20 min. The absorbance was measured at 595 nm using a microplate reader (BioTek, USA), and the biofilm biomass is expressed as a percentage relative to the control group.

For structural analysis of the biofilm, sterile round coverslips were placed at the bottom of 24-well plates before inoculation, and biofilms were cultured as described above. Following the 72 h of anaerobic incubation, the supernatant was discarded, and the wells were rinsed once with PBS to eliminate non-adherent bacteria. The biofilms were stained with SYTO Green nucleic acid stain (Kage Bio, Nanjing, China), diluted 2,000-fold in fresh BHI, and incubated in the dark for 1 h. After washing with PBS, the coverslips were mounted on microscope slides and imaged using a confocal laser scanning microscope (CLSM; AX Confocal Microscope System, Nikon, Japan) equipped with a ×60 oil immersion objective. Z-stack scanning was performed to reconstruct the three-dimensional biofilm architecture (Excitation/Emission = 500/530 nm).

### Quantification of cellular biomass and extracellular polymeric substance (EPS) components in *C. difficile* biofilms exposed to PS-MPs

Sterile round coverslips were placed at the bottom of 24-well plates to establish a biofilm culture system as previously described. Following anaerobic incubation at 37°C for 72 h, the supernatant was discarded, and the biofilms were rinsed three times with phosphate-buffered saline (PBS) to remove non-adherent planktonic bacteria. After air-drying, the coverslips were transferred into centrifuge tubes containing 1 mL of PBS. The samples were sonicated at 37°C for 15 min to disaggregate the biofilm matrix, followed by centrifugation (10,000 rpm, 4°C, 30 min). The resulting supernatant was collected for the quantification of extracellular proteins and polysaccharides within the biofilm matrix, while the pellet was resuspended to determine bacterial cell biomass via the serial dilution plating method. Polysaccharide content was quantified using the phenol-sulfuric acid method, and total protein content was determined using a commercial protein assay kit (Nanjing Jiancheng Bioengineering Institute, China).

### Impact of PS-MPs on motility, AI-2 biosynthesis, and sporulation in *C. difficile*

*C. difficile* (CD) was grown to the mid-exponential phase, adjusted to an OD₆₀₀ of 0.2, and inoculated into BHI medium containing PS-MPs (0, 10, 100, or 400 μg/mL). After 24 h of anaerobic incubation at 37°C, bacterial motility was assessed by stab-inoculating 5 μL of culture into 1/2 BHI semi-solid agar (0.5% agar), and the colony migration diameter was measured after 4 days (19).

#### AI-2 activity

Supernatants from biofilm cultures were collected, centrifuged, and filter-sterilized (0.22 μm). Uninoculated BHI and untreated CD supernatants served as blank and negative controls, respectively. Samples and controls were mixed at a 1:10 volume ratio with a 500-fold diluted overnight culture of *Vibrio campbellii* in fresh AB broth. The mixtures were incubated in a 96-well plate at 30°C with shaking (180 rpm), and relative AI-2 activity was determined by measuring fluorescence.

#### Spore germination

Following a modified protocol (20), CD cultures exposed to PS-MPs for 48 h were centrifuged. The pellets were resuspended in anhydrous ethanol for 1 h to eliminate vegetative cells, then serially diluted and plated to enumerate viable spores.

### Cytotoxicity of cell-free supernatants from PS-MPs-exposed *C. difficile* on intestinal epithelial cells

*C. difficile* was grown to the mid-exponential phase, harvested by centrifugation (4°C, 6,000 rpm, 10 min), and adjusted to an OD₆₀₀ of 0.2. A 50 μL inoculum was added to fresh BHI medium containing PS-MPs (0, 10, 100, or 400 μg/mL) and anaerobically incubated at 37°C for 48 h. Cultures were then centrifuged and filter-sterilized (0.22 μm) to obtain cell-free supernatants (CFS). The CFS was 4-fold diluted with RPMI-1640 or DMEM complete medium (supplemented with 100 IU/mL penicillin and 100 μg/mL streptomycin) and stored at 4°C.

HT-29 and Caco-2 cells at 80% confluence were seeded into 96-well plates at 1 × 10⁴ cells/well (200 μL). After adhesion, the medium was replaced with 200 μL of the diluted CFS. Control wells received 4-fold-diluted BHI medium, while blank wells contained culture medium only (*n* = 6 per group). Following 24 h of incubation (37°C, 5% CO₂), cell viability was assessed using a Cell Proliferation and Toxicity Detection Kit (Albatross, Guangzhou, China).

### Impact of PS-MPs exposure on the adhesion of *C. difficile* to intestinal epithelial cells

*C. difficile* was grown to the mid-exponential phase and inoculated into fresh BHI medium containing PS-MPs (0, 10, 100, or 400 μg/mL) for anaerobic incubation at 37°C for 48 h. Bacterial cells were then harvested, washed three times with PBS, and resuspended in antibiotic-free RPMI-1640 or DMEM to an OD₆₀₀ of 0.2.

HT-29 or Caco-2 monolayers were inoculated with 1 mL of bacterial suspension and incubated anaerobically at 37°C for 2 h. After one PBS wash to remove non-adherent bacteria, cells were detached with 400 μL of 0.25% trypsin for 3 min and then neutralized with 600 μL of medium. The suspensions were then serially diluted, plated, and anaerobically incubated at 37°C for colony enumeration.

### Impact of PS-MPs on the susceptibility of *C. difficile* to vancomycin

The MIC of vancomycin (VAN) against *C. difficile* (CD) was determined via broth microdilution (21). Two-fold serial dilutions of VAN (starting at 512 μg/mL) in BHI were inoculated with bacterial suspension (OD₆₀₀ = 0.2) and incubated anaerobically at 37°C for 48 h; MIC was read via OD₆₀₀ (BioTek H1M).

To evaluate the combined effects of PS-MPs and VAN, a sub-inhibitory concentration of VAN (0.5 μg/mL) was selected based on the MIC results. A 50 μL aliquot of bacterial suspension (OD₆₀₀ = 0.2) was inoculated into 4 mL of BHI medium supplemented with varying PS-MPs concentrations (0, 10, 100, and 400 μg/mL) and 0.5 μg/mL VAN. After anaerobic incubation at 37°C for 48 h, viable cell counts were determined using the plate count method.

### Impact of short- and long-term PS-MPs exposure on antibiotic resistance in *C. difficile*

Seven representative antibiotics used in the clinical treatment of CDI—vancomycin, nitazoxanide, rifaximin, fidaxomicin, ciprofloxacin, tigecycline, and teicoplanin—were selected for antimicrobial susceptibility testing. *C. difficile* (CD) was cultured to the mid-exponential phase, harvested by centrifugation, and resuspended to an OD₆₀₀ of 0.2. A 100 μL inoculum was transferred into 8 mL of fresh BHI medium supplemented with varying concentrations of PS-MPs (0, 10, 100, and 400 μg/mL) and incubated anaerobically at 37°C for 48 h. This culture was designated as Generation 1 (G1). Subsequently, G1 was vortexed evenly, and then, 100 μL of the culture was inoculated into 8 mL of fresh BHI medium supplemented with varying concentrations of PS-MPs for continuous passage. This process was repeated to obtain the 10th generation (G10). Strains from G1 and G10 were harvested to evaluate the impact of short-term and long-term PS-MPs exposure on antibiotic resistance.

Antimicrobial susceptibility testing was performed using a modified method described by Gross et al. (9). Briefly, bacterial suspensions exposed to PS-MPs were inoculated at a 1:100 ratio into BHI medium containing 2-fold serial dilutions of the antibiotics. The tested concentration ranges were as follows: vancomycin (0.0156–32 μg/mL), nitazoxanide (0.0313–32 μg/mL), rifaximin (0.0156–32 μg/mL), fidaxomicin (0.0156–16 μg/mL), ciprofloxacin (0.0625–128 μg/mL), tigecycline (0.0078–16 μg/mL), and teicoplanin (0.0078–16 μg/mL). Each concentration was tested in triplicate. Following incubation at 37°C for 24 h, the OD₆₀₀ was measured using a microplate reader (BioTek, USA). The relative bacterial growth rate was calculated using the following formula: relative growth rate = (OD_*t*_ – OD_0_) / (OD_*c*_ – OD_0_), where OD*_t_* represents the absorbance of the antibiotic-treated well, OD_*c*_ represents the absorbance of the antibiotic-free control well, and OD_0_ represents the absorbance of the blank medium.

### qRT-PCR analysis of key gene expression in *C. difficile* exposed to PS-MPs

Using the same method described above, we collected CD bacteria exposed to PS-MPs for 48 h (G1) and 20 days (G10) via centrifugation for RNA extraction. Total RNA was extracted using the TransZol Up Plus RNA Kit (TransGen Biotech, China). Subsequently, genomic DNA contamination was eliminated, and RNA was reverse transcribed into cDNA using the TransScript One-Step gDNA Removal and cDNA Synthesis Supermix Kit (TransGen Biotech, China). Quantitative real-time PCR (qPCR) was performed to analyze the expression of key genes, categorized as follows: virulence genes (*tcdA*, *tcdB*), quorum-sensing genes (*luxS*, *agrD*), sporulation regulatory genes (*spo0A*, *sigH*), surface protein and adhesion genes (*slpA*, *cwp84*), and antibiotic resistance genes (*tetW*, *gyrA*, *gyrB*). Primer sequences are listed in Table S1. The qPCR reaction was conducted using a two-step amplification protocol: initial pre-denaturation at 94°C for 30 s, followed by 40 cycles of denaturation at 94°C for 5 s and annealing at 60°C for 30 s. A melting curve analysis was performed after amplification to verify the specificity of the amplification products. The 16S rRNA gene served as the internal control. Relative gene expression was quantified using the 2^(−ΔΔCt)^ method (18).

### Statistical analysis

All statistical analyses were performed using GraphPad Prism 8.4.3 (GraphPad Software, LLC, USA). Data are presented as mean ± standard deviation (SD). An ordinary one-way analysis of variance (ANOVA) followed by Tukey’s post hoc test was employed to determine the significance of differences among groups. The analyzed parameters included intracellular ROS and CMP levels in *C. difficile*, biofilm characteristics (thickness, bacterial counts, and extracellular protein and polysaccharide content), cytotoxicity, adhesion capacity, and the expression levels of key genes. Statistical significance is indicated as follows: ns > 0.05; *P  <  0.05; **P  <  0.01; ***P  <  0.001; ****P  <  0.0001.

## RESULTS

### PS-MPs promote *C. difficile* growth and induce oxidative stress and membrane permeability alterations

**Fig 1:**
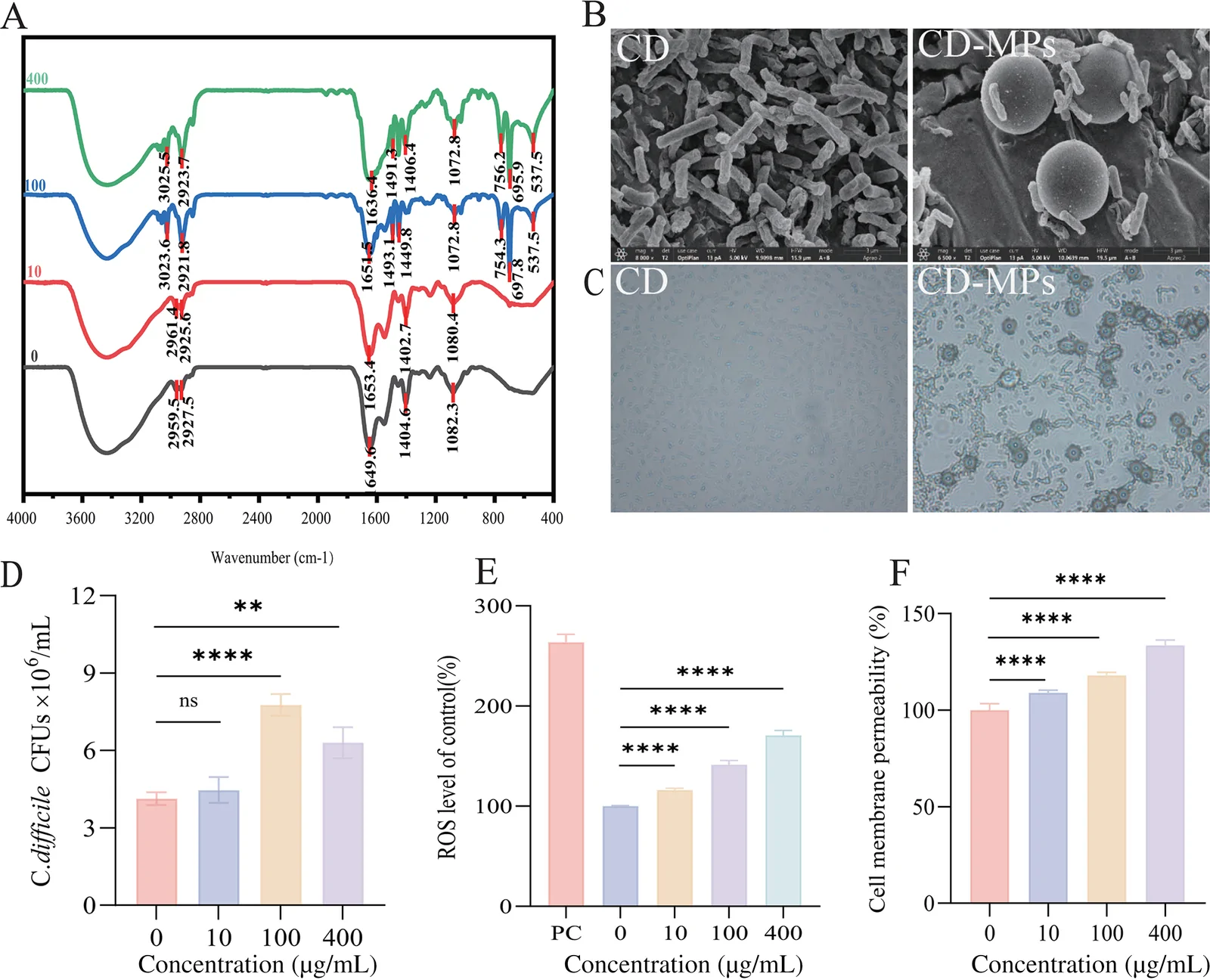
Effects of PS-MPs on CD bacterial morphology and key biological parameters. (**A**) FT-IR spectra of CD bacterial cells after treatment with different concentrations of PS-MPs. (**B**) SEM images of CD bacterial cells after PS-MPs treatment. (**C**) Interaction between CD bacterial cells and PS-MPs using CLSM. (**D**) Effects of different PS-MPs concentrations on CD growth vitality. (**E**) Intracellular ROS levels in CD cells after PS-MPs treatment. (**F**) Cell membrane permeability of CD after PS-MPs treatment. PC: positive control, used to verify the validity of this kit; CD-10: CD treated with 10 μg/mL PS-MPs; CD-100: CD treated with 100 μg/mL PS-MPs; CD-400: CD treated with 400 μg/mL PS-MPs. Significant differences were analyzed using ordinary one-way ANOVA followed by Tukey’s multiple comparisons test. ns > 0.05; *P  <  0.05; **P  <  0.01; ***P  <  0.001; ****P  <  0.0001.

**Fig 2:**
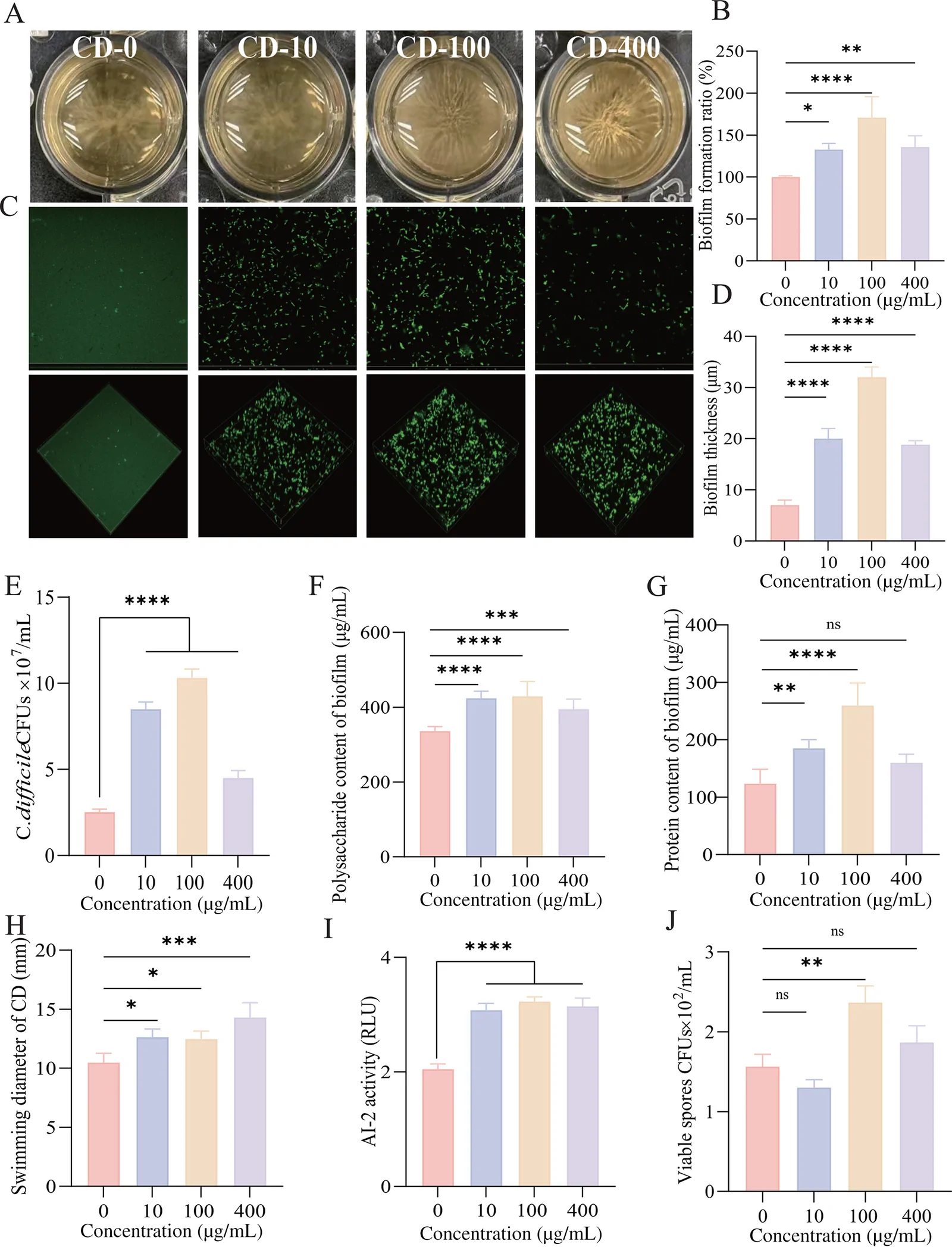
Effects of PS-MPs on CD biofilm formation and motility. (**A**) Biofilm photograph. (**B**) Biofilm content measurement. (**C**) Analysis of biofilm 3D images under laser confocal microscopy (CLSM). Scale bar in laser confocal image: 10 μm. (**D**) Biofilm thickness. (E) CD cell content in biofilms from different treatment groups. (**F**) Extracellular polysaccharide content in biofilms from different treatment groups. (**G**) Extracellular protein content in biofilms from different treatment groups. (**H**) AI-2 content measurement. (**I**) CD motility assay. (**J**) Number of CD spores that germinate after exposure to PS-MPs. CD-0: CD not treated with PS-MPs; CD-10: CD treated with 10 μg/mL PS-MPs; CD-100: CD treated with 100 μg/mL PS-MPs; CD-400: CD treated with 400 μg/mL PS-MPs. Significant differences were analyzed using ordinary one-way ANOVA followed by Tukey’s multiple comparisons test. ns > 0.05; *P  <  0.05; **P  <  0.01; ***P  <  0.001; ****P  <  0.0001.

FT-IR spectral analysis characterized the functional groups of the PS-MPs, revealing distinct absorption peaks at 3025.3–2921.8 cm⁻¹, 1549.7–1402.7 cm⁻¹, and 756.2–537.5 cm⁻¹, which correspond to C-H stretching vibrations (–CH₂, –CH₃), C = C stretching, and C-Br bending vibrations, respectively (Fig. 1A). SEM characterization indicated that the PS-MPs possessed a regular spherical morphology with a uniform diameter of approximately 5 μm (Fig. S1). Notably, SEM coupled with CLSM was employed to characterize the interactions between *C. difficile* (CD) and PS-MPs. The micrographs revealed that abundant CD cells selectively adhered to the surfaces of the PS-MPs, whereas bacterial distribution in the adjacent fields remained notably sparse. These observations demonstrate that CD exhibits a pronounced preferential affinity for PS-MPs (Fig. 1B and 2C). To investigate the biological impact of PS-MPs, CD was exposed to varying concentrations (0, 10, 100, and 400 μg/mL) of the particles. Viable cell counts after 24 h of incubation demonstrated that PS-MPs exposure promoted bacterial growth in a dose-dependent manner (Fig. 1D). Specifically, the 100 μg/mL treatment group exhibited a viable count of 7.7 × 10⁶ CFU/mL, representing a significant increase of 173.88% compared to the control (Fig. 1D). Further physiological analysis revealed that PS-MPs exposure triggered intracellular reactive oxygen species (ROS) accumulation in a dose-dependent manner, peaking at 170.57% of the control level in the 400 μg/mL group (Fig. 1E). Concomitantly, cell membrane permeability was significantly enhanced, increasing to 109.05%, 118.05%, and 133.6% relative to the control in the 10, 100, and 400 μg/mL treatment groups, respectively (Fig. 1F). Collectively, these findings indicate that PS-MPs exposure not only facilitates CD proliferation but also induces oxidative stress and compromises cell membrane integrity, thereby significantly altering the physiological state of the pathogen.

### PS-MPs stimulate extracellular polymeric substance secretion and promote biofilm formation in *C. difficile*

Exposure to PS-MPs significantly promoted biofilm formation in *C. difficile* (CD) (Fig. 2A). Quantitative analysis demonstrated a marked increase in biofilm biomass across all PS-MPs treatment groups (10, 100, and 400 μg/mL), reaching 1.33-, 1.71-, and 1.36-fold that of the control, respectively, with the most pronounced enhancement observed at 100 μg/mL (Fig. 2B). Consistent with the biomass data, three-dimensional confocal laser scanning microscopy (CLSM) revealed a substantial increase in biofilm thickness. Specifically, biofilm thickness increased from 7.00 ± 1.00 µm in the control group to 32.00 ± 2.00 µm in the 100 μg/mL treatment group (Fig. 2D). However, at the highest concentration (400 μg/mL), the biofilm architecture appeared sparse and heterogeneous (Fig. 2C). Given that biofilms are primarily composed of bacterial cells and an extracellular matrix, we quantified these components to elucidate the mechanisms underlying the enhanced biofilm formation. The results indicated that PS-MPs exposure significantly increased bacterial cell density within the biofilm, peaking at 100 μg/mL with a 4.08-fold increase relative to the control (Fig. 2E). Furthermore, PS-MPs treatment significantly stimulated the secretion of extracellular polymeric substances (EPS). The levels of extracellular polysaccharides and proteins exhibited a trend of initial increase followed by a decrease, reaching maximum concentrations of 429.29 μg/mL and 237.23 μg/mL at 100 μg/mL, respectively, corresponding to 1.28- and 1.67-fold increases compared to the control (Fig. 2F and G). Collectively, these findings indicate that PS-MPs enhance the biofilm-forming capacity of CD by promoting bacterial proliferation and stimulating EPS secretion.

### PS-MPs enhance *C. difficile* motility, upregulate AI-2 levels, and promote spore formation

Flagellar-mediated motility serves as a critical determinant for initial adhesion during biofilm formation. Motility assays revealed that PS-MPs exposure significantly enhanced the migratory capacity of *C. difficile* (CD). Specifically, the migration diameter increased from 10.48 mm in the control group to 14.30 mm in the 400 μg/mL treatment group (Fig. 2H). Furthermore, the production of autoinducer-2 (AI-2), a key quorum-sensing molecule regulating biofilm development, was significantly upregulated following PS-MPs exposure, although a distinct concentration-dependent trend was not evident (Fig. 2I). Given that spore germination is the primary risk factor for CDI recurrence, the spore germination yield was further evaluated. Quantitative analysis via plate counting indicated that exposure to PS-MPs promoted spore germination in a concentration-dependent manner, with the highest number of germinating spores observed at 100 μg/mL, reaching 2.37 × 10^2^ CFU/mL (Fig. 2J).

### PS-MPs exposure does not alter the cytotoxicity of *C. difficile* cell-free supernatants against intestinal epithelial cells

**Fig 3:**
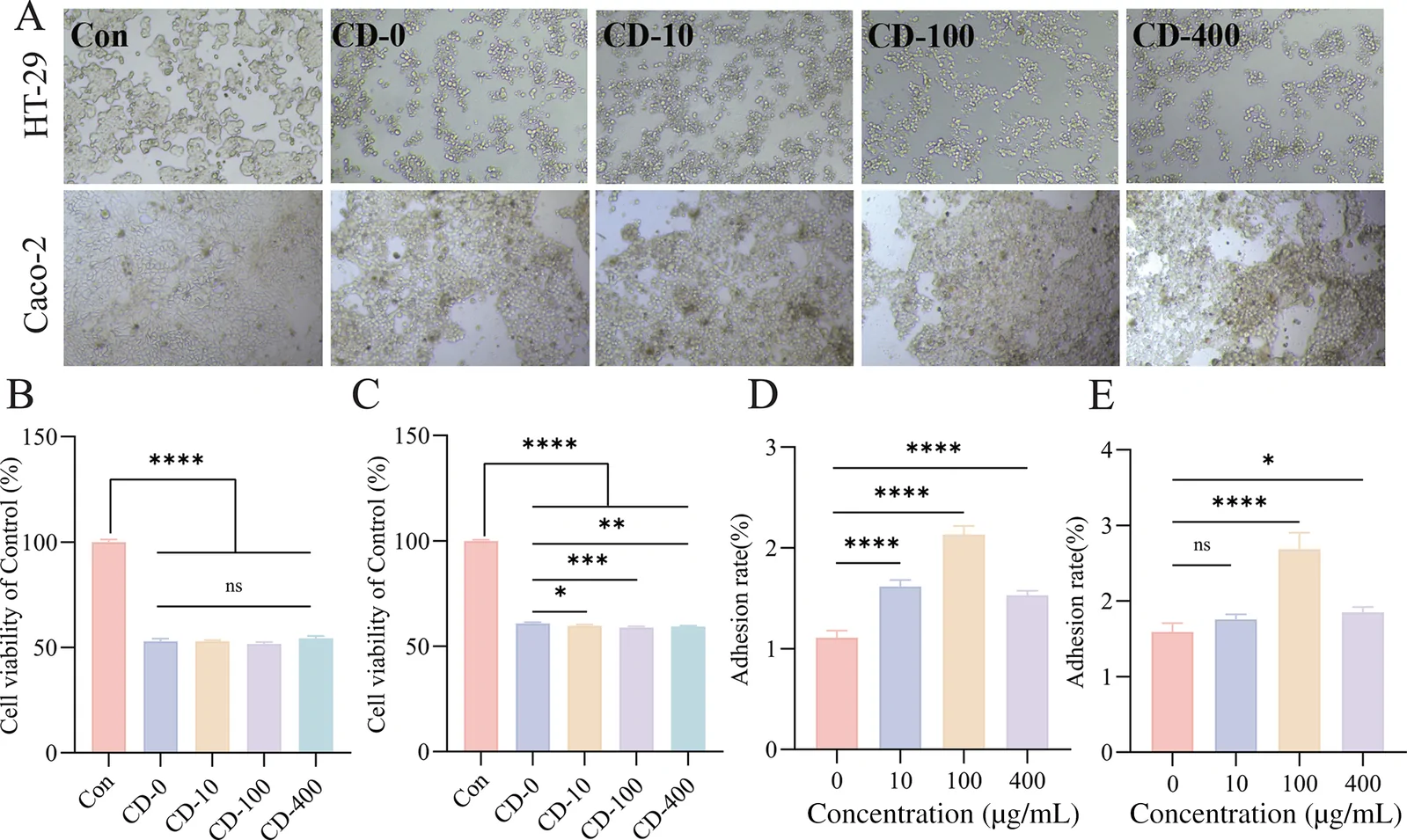
Cytotoxicity of CD against intestinal cells after PS-MPs exposure. (**A**) Morphological effects of cell-free fermentation supernatant from CD on HT-29 and Caco-2 cells after exposure to different PS-MPs concentrations. (**B**) HT-29 cell viability measured by CCK-8 assay after treatment. (**C**) Caco-2 cell viability measured by CCK-8 assay after treatment. (**D**) Adhesion capacity of CD to HT-29 cells after exposure to different concentrations of PS-MPs. (**E**) Adhesion capacity of CD to Caco-2 cells after exposure to different concentrations of PS-MPs. Con: control group treated with blank BHI medium; CD-0: cell-free fermentation supernatant from CD without PS-MPs addition; CD-10: CD cell-free fermentation supernatant after exposure to 10 μg/mL PS-MPs; CD-100: CD cell-free fermentation supernatant after exposure to 100 μg/mL PS-MPs; CD-400: CD cell-free fermentation supernatant after exposure to 400 μg/mL PS-MPs. Significant differences were analyzed using ordinary one-way ANOVA followed by Tukey’s multiple comparisons test. ns > 0.05; *P  <  0.05; **P  <  0.01; ***P  <  0.001; ****P  <  0.0001.

To preliminarily investigate the impact of PS-MPs on the toxigenic potential of *C. difficile* (CD), HT-29 and Caco-2 cells were exposed to cell-free supernatants (CFS) derived from CD cultures treated with varying concentrations of PS-MPs. Inverted microscopy revealed that cells in the control group maintained typical epithelial morphology, characterized by tight intercellular junctions and an orderly arrangement (Fig. 3A). Conversely, treatment with CD CFS for 24 h induced severe morphological damage in both cell lines, as evidenced by marked cell shrinkage, rounding, membrane blebbing, and subsequent fragmentation (Fig. 3A). These observations confirmed the potent cytotoxicity of the CD supernatants against intestinal epithelial cells. Further quantitative assessment using the CCK-8 assay indicated that, compared to the normal control, cell viability was significantly reduced in all groups treated with CD supernatant. However, no significant differences were observed among the groups treated with CFS derived from different PS-MPs concentrations. Specifically, relative to the 0 μg/mL PS-MPs group, the 100 μg/mL PS-MPs group exhibited negligible changes in cell survival rates, with viability shifting slightly from 52.9% to 51.7% in HT-29 cells (Fig. 3B) and from 60.9% to 58.9% in Caco-2 cells (Fig. 3C). These findings suggest that while CD supernatants possess significant cytotoxic activity, PS-MPs exposure does not substantially alter the toxin production capacity of CD or its consequent cytotoxic effects on host cells.

### PS-MPs enhance the adhesion capacity of *C. difficile* to intestinal epithelial cells

Adhesion constitutes a pivotal initial step in *C. difficile* (CD) colonization and subsequent biofilm formation. To elucidate the impact of PS-MPs on the adhesive capacity of CD, adhesion assays were performed using human intestinal epithelial cell lines (HT-29 and Caco-2) following exposure to varying PS-MPs concentrations. The results demonstrated that PS-MPs exposure significantly enhanced the adhesion of CD to both cell lines. Quantitatively, compared to the control group, exposure to 10, 100, and 400 μg/mL PS-MPs increased the adhesion rates of CD to HT-29 cells to 1.46-, 1.92-, and 1.38-fold of the control levels, respectively (Fig. 3D). Similarly, adhesion to Caco-2 cells increased to 1.10-, 1.69-, and 1.16-fold of the control levels, respectively (Fig. 3E). Notably, the most pronounced enhancement was consistently observed at the intermediate concentration of 100 μg/mL. These findings indicate that PS-MPs exposure augments the adhesive properties of CD, exhibiting a peak response at moderate concentrations.

### PS-MPs exposure enhances the resistance of *C. difficile* to vancomycin

The minimum inhibitory concentration (MIC) of vancomycin against *C. difficile* (CD) was first determined using the two-fold serial dilution method, yielding a baseline value of 0.5 μg/mL. To evaluate the impact of PS-MPs on antibiotic resistance, CD was cultured for 48 h in medium containing vancomycin at the MIC level (0.5 μg/mL) supplemented with varying concentrations of PS-MPs, followed by viable plate counting. As anticipated, the vancomycin-only control group (MIC-0) showed no visible colonies, confirming complete growth inhibition at this concentration. In marked contrast, significant bacterial proliferation was observed in all groups co-exposed to vancomycin and PS-MPs (MIC-10, MIC-100, and MIC-400). Quantitative analysis revealed that viable cell counts followed a non-monotonic trend, initially increasing and subsequently decreasing with rising PS-MPs concentrations, peaking in the MIC-100 group at 2.93 × 10⁷ CFU/mL (Fig. S2). Collectively, these findings demonstrate that PS-MPs exposure attenuates the inhibitory efficacy of vancomycin against CD, thereby facilitating bacterial survival under antibiotic stress.

### PS-MPs exposure facilitates the induction of antibiotic resistance in *C. difficile*

Building upon preliminary findings regarding vancomycin tolerance, antimicrobial susceptibility testing was extended to seven clinically relevant antibiotics: vancomycin, nitazoxanide, rifaximin, fidaxomicin, ciprofloxacin, tigecycline, and teicoplanin. The impact of PS-MPs on the development of antibiotic resistance in *C. difficile* (CD) was systematically evaluated using both short-term exposure (48 h) and long-term continuous passaging (20 days) models across varying PS-MPs concentrations (0, 10, 100, and 400 μg/mL) (Fig. S3A). Results from the short-term exposure model indicated that PS-MPs treatment reduced susceptibility to all seven antibiotics to varying degrees, exhibiting a degree of concentration dependence (Fig. S3B through H). Specifically, the median inhibitory concentration (IC₅₀) was elevated in all treatment groups compared to the control (Table 1). The most pronounced shifts in susceptibility were observed for tigecycline and fidaxomicin: at a PS-MPs concentration of 100 μg/mL, the IC₅₀ for tigecycline exhibited a 1.32-fold increase relative to the control, while that for fidaxomicin increased by 1.22-fold. At 400 μg/mL, the IC₅₀ for tigecycline reached 1.48 times that of the control.

**TABLE 1:** Effect of short-term exposure (48 h) to PS-MPs on CD resistance

Antibiotic type	Half inhibitory concentration (IC_50_, 95% CI, μg/mL)			
	Without PS	10 μg/mL PS	100 μg/mL PS	400 μg/mL PS
Vancomycin	0.2802 (0.2707–0.2897) *R*^2^ = 0.9992	0.2893 (0.2779–0.3008) *R*^2^ = 0.9992	0.2972 (0.2815–0.3080) *R*^2^ = 0.9992	0.3079 (0.3015–0.3143) *R*^2^ = 0.9998
Nitazoxanide	2.133 (1.650–2.616) *R*^2^ = 0.983	2.207 (1.750–2.665) *R*^2^ = 0.9832	2.235 (1.997–2.474) *R*^2^ = 0.9955	2.235 (1.981–2.488) *R*^2^ = 0.9943
Rifaximin	0.3053 (0.2115–0.3990) *R*^2^ = 0.9916	0.3053 (0.1899–0.4206) *R*^2^ = 0.9892	0.4119 (0.3405–0.4834) *R*^2^ = 0.9953	0.3708 (0.2771–0.4646) *R*^2^ = 0.9919
Fidaxomicin	0.5493 (0.3075–0.7911) *R*^2^ = 0.9626	0.5773 (0.3175–0.8372) *R*^2^ = 0.9598	0.669 (0.3622–0.9758) *R*^2^ = 0.9493	0.7581 (0.465–1.051) *R*^2^ = 0.9712
Ciprofloxacin	1.648 (1.195–2.102) *R*^2^ = 0.9909	1.662 (1.158–2.166) *R*^2^ = 0.9895	1.789 (1.329–2.248) *R*^2^ = 0.9923	1.809 (1.262–2.356) *R*^2^ = 0.9902
Tigecycline	0.05451 (0.04337–0.06564) *R*^2^ = 0.991	0.06765 (0.05954–0.07576) *R*^2^ = 0.9984	0.07212 (0.06504–0.07920) *R*^2^ = 0.9991	0.08049 (0.06878–0.09220) *R*^2^ = 0.9984
Teicoplanin	0.07274 (0.05869–0.08678) *R*^2^ = 0.9879	0.07371 (0.05965–0.08777) *R*^2^ = 0.986	0.07998 (0.06504–0.07920) *R*^2^ = 0.9907	0.08251 (0.06878–0.09220) *R*^2^ = 0.9934

Long-term exposure (20 days) further exacerbated the evolution of antibiotic resistance in CD (Fig. S3I through O). Compared with the control group, IC₅₀ values were significantly elevated in the long-term exposure groups (Table 2). Critically, except for rifaximin and teicoplanin, resistance levels were further augmented compared to the short-term exposure groups. For instance, in the 100 μg/mL PS-MPs treatment group, the IC₅₀ for vancomycin increased from 0.2972 mg/L after short-term exposure to 0.7182 mg/L after long-term exposure, representing a 2.42-fold escalation. Similarly, the IC₅₀ values for nitazoxanide and ciprofloxacin increased by 4.07-fold and 6.04-fold, respectively, relative to short-term exposure levels. Fidaxomicin exhibited the most dramatic increase, with its IC₅₀ rising from 0.669 mg/L to 4.449 mg/L, representing a 6.65-fold augmentation. Collectively, these findings demonstrate that PS-MPs exposure not only induces short-term tolerance but also significantly accelerates the acquisition of multidrug resistance in CD following prolonged exposure.

**TABLE 2:** Effect of long-term exposure (20 days) to PS-MPs on CD resistance

Antibiotic type	Half inhibitory concentration (IC_50_, 95% CI, μg/mL)			
	Without PS	10 μg/mL PS	100 μg/mL PS	400 μg/mL PS
Vancomycin	0.4523 (0.4123–0.4924) *R*^2^ = 0.9944	0.548 (0.5070–0.5891) *R*^2^ = 0.9961	0.7182 (0.6654–0.7710) *R*^2^ = 0.9962	0.5571 (0.5081–0.6060) *R*^2^ = 0.9953
Nitazoxanide	2.665 (2.489–2.842) *R*^2^ = 0.9931	9.301 (8.564–10.04) *R*^2^ = 0.9916	9.107 (7.811–10.4) *R*^2^ = 0.9972	9.348 (8.595–10.10) *R*^2^ = 0.9867
Rifaximin	0.2645 (0.2498–0.2791) *R*^2^ = 0.999	0.2699 (0.2582–0.2815) *R*^2^ = 0.9964	0.3544 (0.3392–0.3697) *R*^2^ = 0.9973	0.3356 (0.3235–0.3478) *R*^2^ = 0.9982
Fidaxomicin	0.9909 (0.945–1.037) *R*^2^ = 0.9952	4.485 (4.204–4.766) *R*^2^ = 0.9919	4.449 (4.232–4.665) *R*^2^ = 0.9936	4.294 (3.753–4.834) *R*^2^ = 0.98
Ciprofloxacin	2.106 (2.047–2.165) *R*^2^ = 0.9982	8.973 (8.130–9.815) *R*^2^ = 0.9919	10.8 (9.645–11.95) *R*^2^ = 0.9869	8.433 (7.318–9.547) *R*^2^ = 0.9861
Tigecycline	0.1162 (0.1026–0.1299) *R*^2^ = 0.9913	0.1558 (0.1380–0.1737) *R*^2^ = 0.9956	0.1758 (0.0.1490–0.2025) *R*^2^ = 0.9922	0.1541 (0.1385–0.1698) *R*^2^ = 0.9962
Teicoplanin	0.06364 (0.05812–0.06915) *R*^2^ = 0.9866	0.06698 (0.06289–0.07107) *R*^2^ = 0.9919	0.07085 0.06494–0.07675) *R*^2^ = 0.9872	0.07186 (0.06671–0.07702) *R*^2^ = 0.9895

### PS-MPs upregulate the expression of virulence and antibiotic resistance genes in *C. difficile*

**Fig 4:**
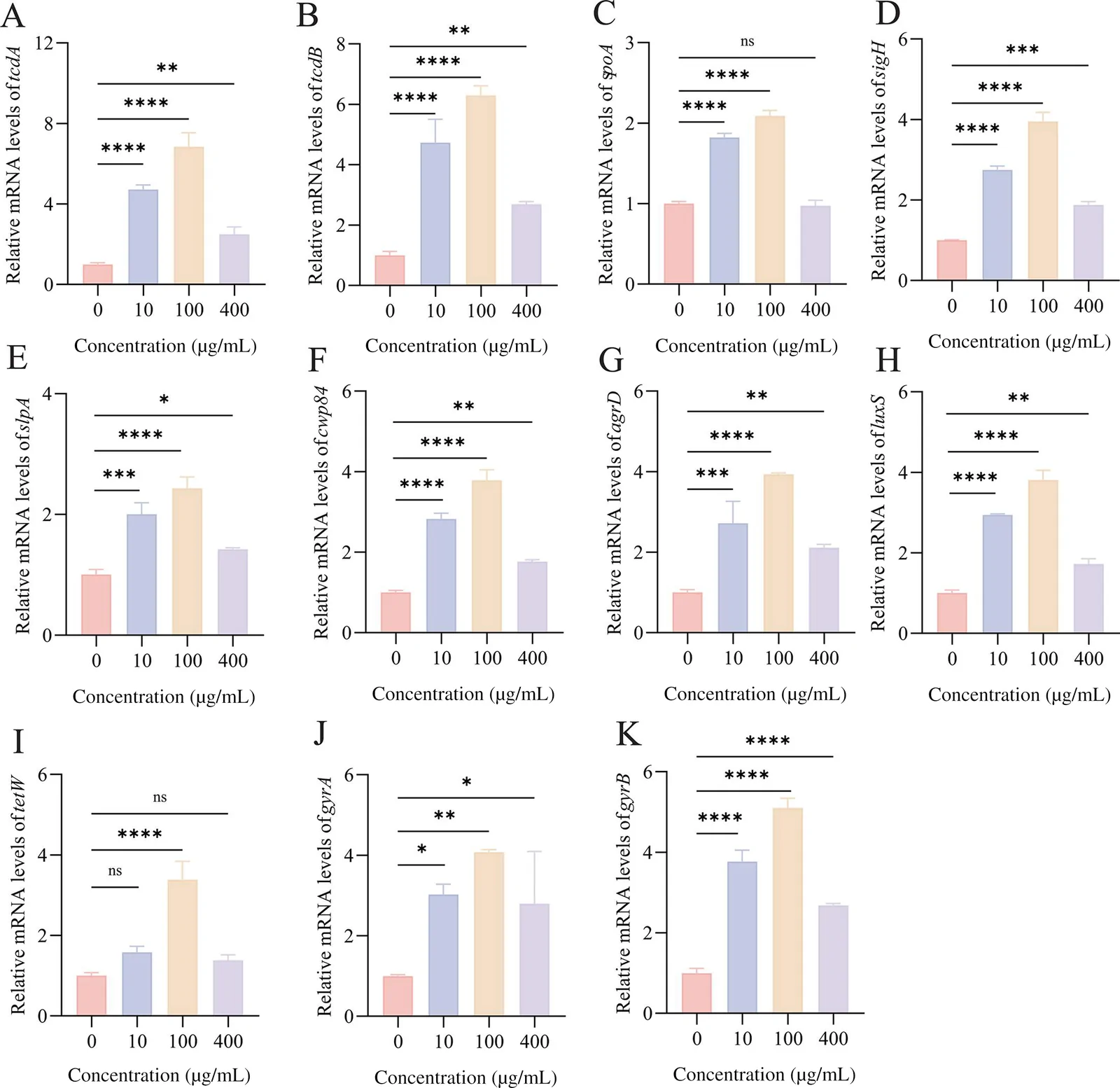
Gene expression of CD after 48 h of exposure to PS-MPs. (**A–K**) Expression levels of *tcdA*, *tcdB*, *spoA*, *sigH*, *slpA*, *cwp84*, *agrD*, *luxS*, *tetW*, *gyrA*, and *gyrB*, respectively. Significant differences were analyzed using ordinary one-way ANOVA followed by Tukey’s multiple comparisons test. ns > 0.05; *P  <  0.05; **P  <  0.01; ***P  <  0.001; ****P  <  0.0001.

To elucidate the molecular mechanisms underlying the impact of PS-MPs on *C. difficile* (CD) pathogenicity and antibiotic resistance, qRT-PCR was performed to quantify the relative mRNA expression levels of key genes after 48 h of exposure (Fig. 4). The results indicated that PS-MPs exposure significantly modulated the transcriptional profile of CD, characterized by a non-monotonic dose-response relationship where upregulation typically peaked at the intermediate concentration (100 μg/mL). Specifically, the expression of virulence genes *tcdA* and *tcdB* was significantly upregulated, reaching 6.85- and 6.29-fold of control levels at 100 μg/mL, respectively (Fig. 4A and B). Similarly, the sporulation regulatory genes *spo0A* and *sigH* exhibited comparable trends, peaking at 2.09- and 3.95-fold, respectively, suggesting that PS-MPs may exacerbate the risk of infection recurrence by promoting sporulation (Fig. 4C and D). Regarding colonization, the adhesion-associated genes *slpA* and *cwp84* were significantly induced. Notably, *cwp84* expression reached 3.79-fold of the control at 100 μg/mL (Fig. 4E and F). Furthermore, key quorum-sensing (QS) system genes, *agrD* and *luxS*, were significantly activated, reaching 3.93- and 3.81-fold increases at 100 μg/mL, respectively (Fig. 4G and H). Corroborating the phenotypic resistance data, antibiotic resistance-associated genes (*tetW*, *gyrA*, and *gyrB*) showed significant upregulation within the 10–100 μg/mL range (Fig. 4I through 4K). Interestingly, at the highest concentration (400 μg/mL), the magnitude of upregulation generally declined compared to the 100 μg/mL group. The expression of certain genes (e.g., *spo0A*, *tetW*) was significantly attenuated, implying that high-dose PS-MPs may induce physiological stress or exert inhibitory effects on bacterial metabolism. In summary, PS-MPs exposure induces differential upregulation of genes involved in virulence, sporulation, adhesion, and resistance, with the most potent inductive effect observed at the intermediate concentration (100 μg/mL).

### Long-term PS-MPs exposure exacerbates the upregulation of sporulation and antibiotic resistance genes in *C. difficile*

**Fig 5:**
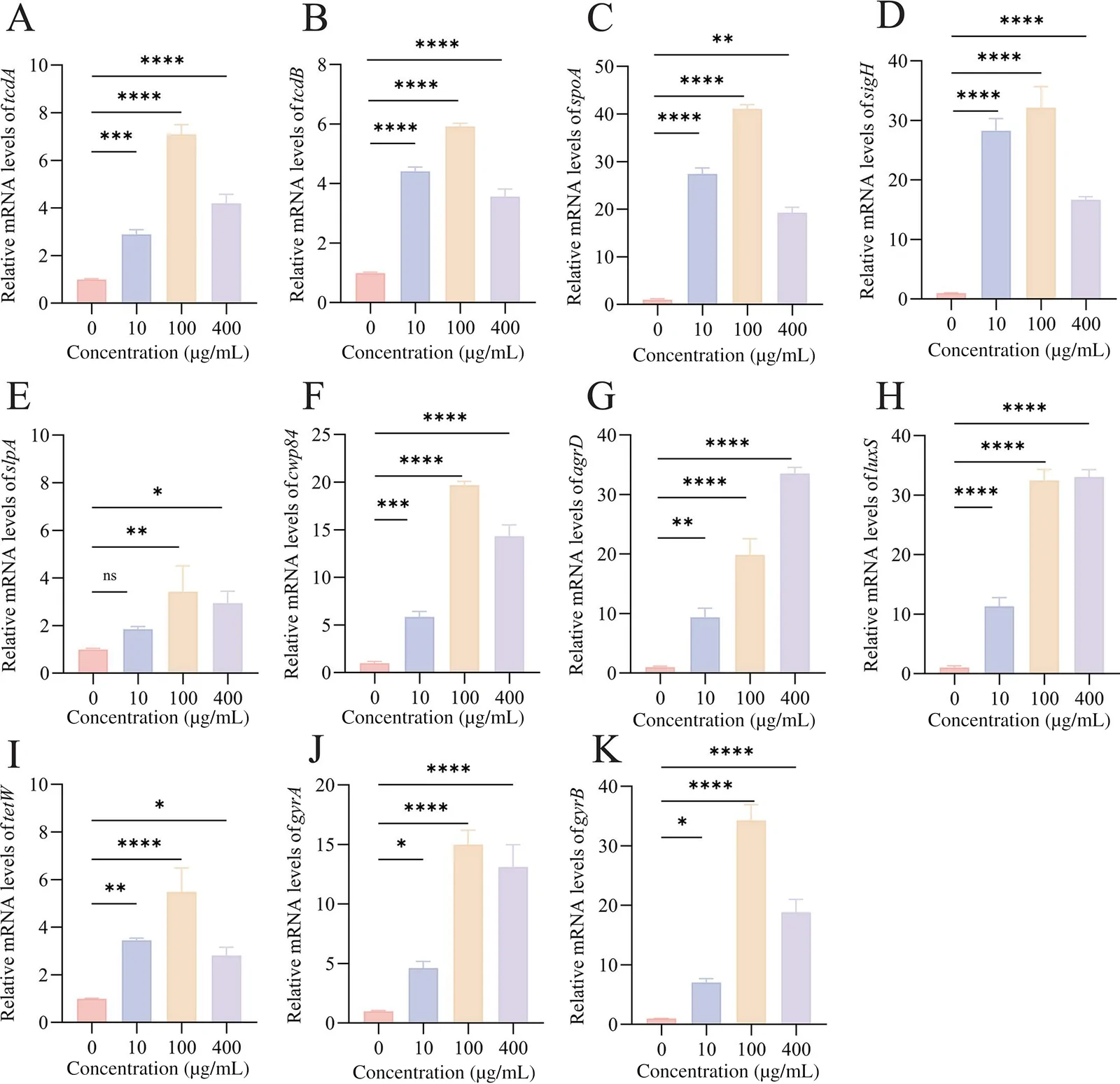
Gene expression of CD after 20 days of exposure to PS-MPs. (**A–K**) Expression levels of *tcdA*, *tcdB*, *spoA*, *sigH*, *slpA*, *cwp84*, *agrD*, *luxS*, *tetW*, *gyrA*, and *gyrB*, respectively. Significant differences were analyzed using ordinary one-way ANOVA followed by Tukey’s multiple comparisons test. ns > 0.05; *P  <  0.05; **P  <  0.01; ***P  <  0.001; ****P  <  0.0001.

Given the pronounced exacerbation of antibiotic resistance in *C. difficile* (CD) following prolonged PS-MPs exposure, we employed qRT-PCR to evaluate the relative mRNA expression levels of key genes after 20 days of exposure, aiming to elucidate the underlying molecular mechanisms (Fig. 5). The results demonstrated that chronic exposure triggered a widespread and significant upregulation of multiple functional genes compared to the control group. Critically, when compared to short-term exposure, prolonged exposure induced a further dramatic amplification in the expression of genes governing sporulation (*spo0A* and *sigH*), adhesion (*cwp84*), quorum sensing (*agrD* and *luxS*), and antibiotic resistance (*gyrA* and *gyrB*). Specifically, at the 100 μg/mL concentration, the sporulation regulators *spo0A* and *sigH* were upregulated to 41.06- and 32.17-fold of the control levels, respectively (Fig. 5C and D); the adhesion-related gene *cwp84* reached 19.68-fold (Fig. 5F); the quorum-sensing genes *agrD* and *luxS* peaked at 19.86- and 32.46-fold, respectively (Fig. 5G and H); and the resistance-associated genes *gyrA* and *gyrB* were elevated to 15.00- and 34.28-fold, respectively (Fig. 5J and K). Collectively, these qRT-PCR findings reveal that PS-MPs exposure induces a profound differential upregulation of diverse functional genes in CD. This transcriptional activation is vastly intensified by long-term exposure, providing a robust molecular basis for the observed phenotypic enhancements in biofilm formation, sporulation, and antibiotic resistance.

## DISCUSSION

*Clostridioides difficile* infection (CDI) has emerged as a critical global public health crisis (6). Recent evidence underscores the pervasive contamination of the food chain with *C. difficile* (CD) spores, highlighting a significant risk of human exposure and infection via foodborne transmission (15, 22). Despite clinical interventions such as antibiotic therapy, probiotic administration, and fecal microbiota transplantation (FMT), the global incidence and recurrence rates of CDI remain persistently high (23). While biofilm formation and the acquisition of antibiotic resistance are established drivers of CDI pathogenesis and recurrence, the potential modulatory role of environmental factors—distinct from antibiotic selection pressure—remains underexplored. Notably, previous studies have demonstrated that microplastics (MPs) exposure can significantly enhance the biofilm-forming capacity and antibiotic resistance of pathogens, such as *Escherichia coli*, *Pseudomonas aeruginosa*, and *Vibrio cholerae*, thereby elevating infection risks (9–12). However, the interaction between MPs and CD remains poorly understood. Building upon this context, the present study investigates the interaction between MPs and CD. Humans are exposed to a broad spectrum of MPs sizes, among which the 1–10 μm fraction is the most frequently detected in major dietary sources (e.g., drinking water, seafood, and salt) and poses the greatest toxicological significance (24). Specifically, 5 μm serves as a representative benchmark size extensively utilized in studies concerning intestinal toxicology and host-microbe interactions (25–27). Crucially, while 5 μm MPs possess the theoretical capacity to traverse the intestinal epithelium, their actual translocation efficiency is inherently low. Consequently, the vast majority of ingested 5 μm MPs are retained within the intestinal lumen and mucus layer. This preferential luminal retention provides a critical spatial basis for direct physical and physiological interactions between MPs and CD, an obligate anaerobe predominantly residing in this mucosal microenvironment.

Regarding exposure dosages, MPs have been detected in human organs and body fluids ranging from 0.43 to 275.78 μg/g, with median fecal concentrations reaching up to 345 μg/g (28). Furthermore, systematic reviews and meta-analyses indicate that 10 μg/mL represents the lowest environmentally relevant concentration capable of inducing detectable cytotoxicity in human cells (29). Integrating this scientific evidence regarding both particle size and concentration to optimally simulate realistic exposure scenarios, this study selected 5 μm polystyrene microplastics (PS-MPs) as the model pollutant. By establishing low-, medium-, and high-dose groups (10, 100, and 400 μg/mL, respectively), we aim to systematically elucidate the impact of PS-MP exposure on the core biological characteristics of CD—including biofilm formation, sporulation, and toxin production—as well as its antibiotic resistance. Ultimately, this work seeks to assess the potential risk of CDI exacerbation in the context of widespread microplastic exposure.

The modulatory effects of microplastics on bacterial growth have been well documented. For instance, exposure to micro- and nanoplastics has been shown to significantly enhance the cell survival rate of *Halomonas alkaliphila*, characterized by growth promotion at low concentrations and inhibition at high concentrations (30). Consistent with these findings, the present study demonstrated that low-to-moderate concentrations of PS-MPs (10–100 μg/mL) did not inhibit CD growth but, conversely, stimulated its proliferation. However, the toxic effects of microplastics on bacteria are well established, primarily attributed to the induction of intracellular oxidative stress (31). Indeed, our SEM and FT-IR analyses confirmed that PS-MPs exposure alters the cell membrane composition of CD. Furthermore, a concentration-dependent increase in intracellular ROS levels and membrane permeability was observed, indicative of heightened cytotoxicity at higher microplastic concentrations. To reconcile this biphasic dose response, where growth promotion persists alongside signs of toxicity, Zhang et al. provided a key insight: moderate increases in ROS levels can stimulate bacterial proliferation, whereas excessive ROS exerts inhibitory effects (32). This phenomenon aligns with findings by Shen et al., who reported that PS-MPs and norfloxacin elevated ROS levels in *Escherichia coli* to a moderate range (approximately 140% of control), potentially enhancing metabolic activity and thereby facilitating cell proliferation (17). Generally, the generation of oxidative stress triggers a cascade of intracellular defense responses, such as alterations in membrane permeability and the secretion of extracellular polymeric substances (EPS) (30). Based on the observation that PS-MPs exposure increased CD viability while concurrently enhancing membrane permeability and intracellular ROS levels, we hypothesize that the interaction between CD and PS-MPs induces oxidative stress. To mitigate this stress, the bacteria activate defense mechanisms—including promoting EPS secretion and biofilm formation—which may inadvertently sustain and enhance bacterial proliferation under specific exposure conditions.

Biofilm formation constitutes a critical defense strategy for bacteria against environmental stressors. Indeed, stimuli such as antibiotics and nanoparticles are well-documented inducers of its development (33, 34). In this study, PS-MPs exposure significantly augmented the biofilm-forming capacity of CD and increased biofilm thickness. These findings align with Gross et al., who reported that microplastics facilitate the formation of thick, dense biofilms in *E. coli* (9). Notably, however, a decline in both biofilm biomass and thickness was observed at the highest PS-MPs concentration (400 μg/mL). Compositional analysis revealed that the levels of key structural components—including extracellular proteins, polysaccharides, and bacterial cell density—increased significantly following PS-MPs exposure, yet exhibited a downward trend at high concentrations. This pattern paralleled the variations in total biofilm biomass, suggesting that excessive stress induced by high PS-MPs concentrations may impede the synthesis or accumulation of matrix components. Flagellar-mediated motility serves as the initial step in biofilm formation, while the quorum-sensing (QS) system functions as the central regulatory mechanism governing biofilm development and virulence expression (35). While Wang et al. noted that nanoplastics enhance biofilm formation in *Pseudomonas aeruginosa* and *E. coli* by modulating the QS system (36), the present study found that PS-MPs enhanced CD motility alongside elevated levels of the QS signaling molecule AI-2. Concurrently, bacterial adhesion to Caco-2 and HT-29 cells was significantly enhanced, underscoring the heightened pathogenic potential of CD under PS-MPs stimulation. qRT-PCR analysis provided molecular evidence for these phenotypic alterations, demonstrating significant upregulation of virulence genes (*tcdA*, *tcdB*), QS-related genes (*agrD*, *luxS*), and adhesion-related genes (*slpA*, *cwp84*). Collectively, these findings suggest that CD activates its QS system in response to PS-MPs exposure, thereby synergistically regulating biofilm formation and virulence factor secretion to adapt to this environmental stress.

The interaction between microplastics and bacterial biofilms not only amplifies pathogenicity but may also exacerbate antibiotic resistance (37). Given the established correlation between antibiotic resistance in CD and its biofilm formation or sporulation capabilities (38), this study evaluated the impact of PS-MPs on the resistance spectrum of this pathogen. Our findings revealed that exposure to polystyrene microplastics significantly increased spore germination and enhanced resistance to clinically relevant antibiotics, with prolonged exposure yielding more pronounced effects. qRT-PCR analysis demonstrated significant upregulation of resistance-associated genes (*gyrA*, *gyrB*, and *tetW*), providing molecular evidence underpinning the observed phenotypic resistance. These observations align with previous reports; for instance, Gross et al. demonstrated that microplastics facilitate antibiotic resistance in *E. coli* by promoting biofilm formation (9), while Tang et al. reported similar outcomes in *Acinetobacter johnsonii* AC15 exposed to nano-polystyrene (39). Mechanistically, mature biofilms can impede antibiotic penetration, thereby shielding resident bacteria (40). Additionally, microplastics possess a substantial adsorptive capacity for antibiotics, potentially reducing drug bioavailability by hindering their interaction with bacterial cells (41). Consequently, the elevated antibiotic resistance observed in this study may be attributed to a combination of enhanced biofilm/spore formation and the adsorption of antibiotics by PS-MPs. Further investigation is required to elucidate whether these mechanisms operate independently or synergistically. Although this study provides *in vitro* evidence that PS-MPs enhance the pathogenicity of CD, certain limitations remain. Future research should validate these findings *in vivo* using animal models that simulate the complex gut microenvironment. Furthermore, it is critical to assess the risks associated with microplastics of varying physicochemical properties (e.g., chemical composition, size, surface charge) and their interactions with co-existing pollutants in realistic settings. Subsequent studies will focus on developing physiological models of the human intestine to systematically elucidate the broader implications of microplastic exposure on CD colonization, toxin expression, and overall infectivity.

### Conclusions

In summary, this study systematically elucidates the mechanisms by which polystyrene microplastics (PS-MPs) exposure exacerbates the pathogenicity of *C. difficile* (CD). Through the coordinated upregulation of virulence, quorum sensing, sporulation, adhesion, and antibiotic resistance genes, PS-MPs drive the excessive secretion of extracellular polymeric substances and robust biofilm formation. This transcriptional and structural remodeling consequently enhances CD motility, adhesion capacity, toxin production, sporulation efficiency, and antibiotic resistance. Notably, these pathogenic enhancements exhibit distinct concentration- and time-dependent patterns, peaking at 100 μg/mL and being further amplified by prolonged exposure. Collectively, these systematic alterations in core biological characteristics suggest that PS-MPs exposure may substantially augment the intestinal colonization capacity, infection persistence, and relapse potential of CD.

## Data Availability

The data that support the findings of this study are available in Figshare at https://doi.org/10.6084/m9.figshare.31771042.v2.

## SUPPLEMENTAL MATERIAL

Supplemental material aem.01168-26-s0001.docx Fig. S1 to S3; Table S1.

ASM does not own the copyrights to Supplemental Material that may be linked to, or accessed through, an article. The authors have granted ASM a non-exclusive, world-wide license to publish the Supplemental Material files. Please contact the corresponding author directly for reuse.
