# Supplementary material for: Polystyrene microplastics facilitate Clostridioides difficile biofilm formation and attenuate antibiotic susceptibility
Source: Appl Environ Microbiol. 2026 Jul 31;92(8):e01168-26. doi: 10.1128/aem.01168-26 (PMC13488320; doi:10.1128/aem.01168-26)
Supplement: Supplementary file 1 [file aem.01168-26-s0001.docx]

**Supplementary Material**

**Figure S1. Observation of PS-MPs.** A. SEM observation of PS-MPs; B. Determination of particle size for PS-MPs.

**Figure S2. Survival rate of CD under vancomycin in the PS-MPs environment.** Control: Represents the natural growth state of CD under normal conditions; the MIC-0 group is the treatment group to which the minimum inhibitory concentration (MIC) of vancomycin was added but no PS-MPs; the MIC-10, MIC-100, and MIC-400 groups are combination treatment groups to which 10, 100, and 400 μg/mL of PS-MPs, respectively, were added to the MIC concentration of vancomycin. Significant differences were analyzed using Ordinary one-way ANOVA followed by Tukey’s multiple comparisons test in Fig. S2. **p < 0.01; ***p < 0.001; ****p < 0.0001.

**Figure S3. Effect of PS-MPs Exposure on CD Antibiotic Resistance.** A. Experimental workflow for CD antibiotic susceptibility testing; B–H. Concentration-response relationships of CD under seven antibiotics after short-term PS-MPs exposure; I–O. Concentration-response relationships of CD under seven antibiotics after long-term PS-MPs exposure. Control: CD grown under normal conditions without PS-MPs.

**Table S1** Primer sequences used in this study.

| Target gene | 5’Primer | 3’Primer | Ref |
| --- | --- | --- | --- |
| *16S rRNA* | AGAGTTTGATCCTGGCTCAG | GGTTACCTTGTTACGACTT | (1) |
| *tcdA* | CAACACCTTAACCCAGCCATA | AGAGTTTTCTGCGGTAGCTGA | (1) |
| *tcdB* | ATCTGGAGAATGGAAGGTGGT | TGATGGTGCTGAAAAGAAGTG | (1) |
| *Spo0A* | AGCGCAATAAATCTAGGAGCAGA | TGGTCTAGGTTTTGGCTCAACT | (1) |
| *sigH* | GTTGGTAGCAAAAGAAAAAAGTTATGAG | GTACTCTAGTGCTATTTTATCCCCTTCAC | (2) |
| *slpA* | AATGATAAAGCATTTGTAGTTGGTG | TATTGGAGTAGCATCTCCATC | (3) |
| *luxS* | GTGTACTTGATGGAGTAAAGGGAGA | TTCTACATCCCATTGGAGATAAGTC | (4) |
| *cwp84* | TGGGCAACTGGTGGAAAATA | TAGTTGCACCTTGTGCCTCA | (4) |
| *agrD* | TTTGCTAGCTCATTGGCACTT | GATTGCTGATTTCTTTGGGTACTT | (5) |
| *gyrA* | TTTAAAGCCAGTTCATAG | GAACCAAAGTTACCATG | (6) |
| *gyrB* | AGTTGATGAACTGGGGTCTT | TCAAAATCTTCTCCAATACCA | (7) |
| *tetW* | CATCTCTGTGATTTTCAGCTTTTCTCTCCC | AGTCTGTTCGGGATAAGCTCTCCGCCG | (8) |

**Reference**

1.	Yang J, Rui W, Zhong S, Li X, Liu W, Meng L, Li Y, Huang H. 2024. Symbiotic biofilms formed by Clostridioides difficile and bacteroides thetaiotaomicron in the presence of vancomycin. Gut Microbes 16:2390133.

2.	Karlsson S, Burman LG, Åkerlund T. 2008. Induction of toxins in Clostridium difficile is associated with dramatic changes of its metabolism. Microbiology (Reading) 154:3430-3436.

3.	Denève C, Deloménie C, Barc MC, Collignon A, Janoir C. 2008. Antibiotics involved in Clostridium difficile-associated disease increase colonization factor gene expression. J Med Microbiol 57:732-738.

4.	Yun B, Oh S, Griffiths MW. 2014. Lactobacillus acidophilus modulates the virulence of Clostridium difficile. J Dairy Sci 97:4745-58.

5.	Tijerina-Rodríguez L, Villarreal-Treviño L, Baines SD, Morfín-Otero R, Camacho-Ortíz A, Flores-Treviño S, Maldonado-Garza H, Rodríguez-Noriega E, Garza-González E. 2019. High sporulation and overexpression of virulence factors in biofilms and reduced susceptibility to vancomycin and linezolid in recurrent Clostridium [Clostridioides] difficile infection isolates. PLoS One 14:e0220671.

6.	Ackermann G, Tang YJ, Kueper R, Heisig P, Rodloff AC, Silva J, Jr., Cohen SH. 2001. Resistance to moxifloxacin in toxigenic Clostridium difficile isolates is associated with mutations in gyrA. Antimicrobial Agents and Chemotherapy 45:2348-2353.

7.	Dridi L, Tankovic J, Burghoffer B, Barbut F, Petit JC. 2002. gyrA and gyrB mutations are implicated in cross-resistance to ciprofloxacin and moxifloxacin in Clostridium difficile. Antimicrobial Agents and Chemotherapy 46:3418-3421.

8.	Fry Pamela R, Thakur S, Abley M, Gebreyes Wondwossen A. 2012. Antimicrobial Resistance, Toxinotype, and Genotypic Profiling of Clostridium difficile Isolates of Swine Origin. Journal of Clinical Microbiology 50:2366-2372.
